# Synthesis of MPEG-b-PLLA Diblock Copolymers and Their Crystallization Performance with PDLA and PLLA Composite Films

**DOI:** 10.3390/ma17092105

**Published:** 2024-04-29

**Authors:** Wenjing Wu, Weixin Wu, Mingwei Guo, Ruizhe Wang, Xuanxuan Wang, Qinwei Gao

**Affiliations:** 1 College of Chemical Engineering, Nanjing Forestry University, Nanjing 210037, China; jingwenwu1999@njfu.edu.cn (W.W.); 17863926658@163.com (W.W.); wangruizhe_ecust@126.com (R.W.); 15098299627@163.com (X.W.); 2 College of Chemistry and Chemical Engineering, Nanjing University, Nanjing 210023, China; guomw199876@163.com; 3 Jiangsu Key Laboratory for the Chemistry and Utilization of Agricultural and Forest Biomass, Nanjing Forestry University, Nanjing 210037, China

**Keywords:** polylactic acid, polyethylene glycol, diblock copolymer, stereocomposite crystals, homocrystals

## Abstract

Methoxy poly(ethylene glycol)-block-poly(L-lactide) (MPEG-b-PLLA) has a wide range of applications in pharmaceuticals and biology, and its structure and morphology have been thoroughly studied. In the experiment, we synthesized MPEG-b-PLLA with different block lengths using the principle of ring-opening polymerization by controlling the amount of lactic acid added. The thermodynamic properties of copolymers and the crystallization properties of blends were studied separately. The crystallization kinetics of PDLA/MPEG-b-PLA and PLLA/MPEG-b-PLA composite films were studied using differential scanning calorimetry (DSC). The results indicate that the crystallization kinetics of composite films are closely related to the amount of block addition. The crystallinity of the sample first increases and then decreases with an increase in MPEG-b-PLLA content. These results were also confirmed in polarized optical microscope (POM) and wide-angle X-ray diffraction (WAXD) tests. When 3% MPEG-b-PLLA was added to the PDLA matrix, the blend exhibited the strongest crystallization performance.

## 1. Introduction

The fermentation of agricultural and sideline products produces lactic acid, and polylactic acid (PLA) is polymerized from lactic acid monomers [1,2]. PLA can be naturally degraded and is widely used in the field of biomedicine [3]. The crystallization performance has a significant impact on the molding and processing of polymers [4,5], ultimately affecting the performance of products. Stereocomplex (SCs) crystallization is a common phenomenon in polymer crystallization [6], where polymer chains that are stereoisomeric can undergo SCs crystallization in both blend systems and block copolymers [7]. The SCs crystallization of PLA is the most representative of the SCs crystallization system. In SCs-PLA, there are strong intermolecular interactions (hydrogen bonds) [8,9] between the molecular chains, which can form tight packing [10]. This unique structure often makes materials with stereocomposite crystallization have higher melting points [11,12] and better crystallization ability [12], heat resistance [13], mechanical properties [14], and chemical stability [15].

The melting temperature (T_m_) of PLA is 150~170 °C, and its glass transition temperature (T_g_) is 60 °C [16]. However, for traditional PLA materials, the disadvantages of low usage temperature and poor durability limit their application. Block copolymerization is an effective method for improving the performance of a single PLA material. The addition of PEG components can improve the hydrophilicity of PLA [17] while improving its resistance to degradation [18] and other physical properties [19]. Therefore, PEG-PLA has many applications in the field of drug release [20]. PEG-PLA block copolymers can form hydrogels [21], micelles [22], nanoparticles [23], etc. They have been used in biopharmaceutical fields such as controlled drug release and tissue engineering [24,25]. For example, morphology [26], self-organization [27], crystal structure [28], and crystallization rate [29,30] have been discussed in a variety of situations. The crystallization characteristics of one segment in block copolymers containing two crystal segments are influenced by the composition, structure, and size of the other segments [31]. Researching these impacts aids in enhancing the comprehension of crystallization patterns exhibited by block copolymers. In our previous work, we synthesized a block copolymer with a wide range of PLLA/PDLA molecular weights to study crystallization and melting behaviors [32,33,34]. However, a comprehensive investigation into the crystallization kinetics across various block lengths has not been conducted, a factor that holds importance in the regulation of performance and comprehension of crystallization tendencies exhibited by block copolymers.

This study synthesized MPEG-b-PLLA diblock copolymers with different PEG contents. Then, the crystallization behavior of MPEG-b-PLLA and its composite films with PDLA and PLLA was analyzed using differential scanning calorimetry (DSC). Simultaneously, these samples were isothermally crystallized at different times via a polarized optical microscope (POM). The results indicate that the crystallization behavior of block polymer MPEG-b-PLLA depends on its length; in contrast, for composite membrane samples, it depends on the amount of MPEG-b-PLLA block polymer added. With an increase in MPEG_2_-PLLLA_2_, the crystallization rate increases first and then decreases.

## 2. Materials and Methods

### 2.1. Synthesis of MPEG-b-PLLA Diblock Copolymers

The raw materials required for the experiment included tin(II)_2_-ethylhexanoate (Sn(Oct)_2_, >99.9%), toluene (99.8%, anhydrous), ethyl ether (99.8%, anhydrous), CHCl_3_ (density 1.47–1.48 g/mL), L-lactic acid monomer, and MPEG (M_n_ = 2000 g/mol, Đ = 1.08).

**Figure 1:**
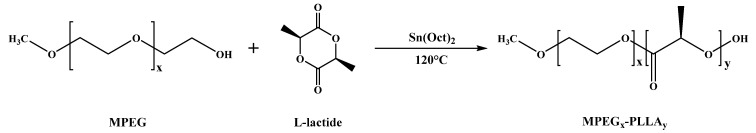
Synthesis of MPEG-b-PLLA.

The synthesis route is shown in Figure 1. PLLA was synthesized via the ring-opening polymerization of L-lactide, following methods outlined in the existing literature [35,36]. Using Sn (Oct)_2_ as the catalyst and PEG as the initiator, MPEG-b-PLLA block copolymers were synthesized via the ring-opening polymerization (ROP) of PLA intermediate lactide in a toluene environment at 120 °C for 12 h. The copolymers were isolated via precipitation in diethyl ether. The unreacted raw materials and impurities were consumed during treatment with ethanol. MPEG and PLLA blocks were prepared with different molecular weights, and they were named MPEG_2_-PLLA_1_, MPEG_2_-PLLA_2_, MPEG_2_-PLLA_3_, and MPEG_2_-PLLA_4_. Numeric subscripts represent the proportion of each component in the polymer.

### 2.2. Solution-Cast Composite Films

PLA composite films were produced using a technique known as solution casting, with certain adjustments made to the process, as outlined in a prior scholarly article [37]. We chose MPEG_2_-PLLA_2_. Various loadings (1, 2, 3, 5 and 10 wt %) of MPEG-b-PLLA were used as fillers in the PDLA matrix to prepare PDLA/MPEG-b-PLLA and PLLA/MPEG-b-PLLA films, which were named PDLA-1, PDLA-2, PDLA-3, PDLA-5, and PDLA-10. Similarly, the same operations were applied with PLLA as a matrix, and these films were named PLLA-1, PLLA-2, PLLA-3, PLLA-5, and PLLA-10. A specified amount of MPEG-b-PLLA copolymer was mixed with 50 milliliters of CHCl_3_ and stirred magnetically at 500 rpm for 2 h. The solution was filtered to remove all insoluble impurities. After filtration, the filtrate was transferred to a conical flask, PLLA or PDLA was added, and mixing was carried out thoroughly for 2 h via magnetic stirring. After mixing, the solution was poured into a suitably sized mold and left to stand in a fume hood for 12 h. Then, the resulting film was dried in a vacuum oven at 40 °C for 12 h to eliminate any remaining solvents. Subsequently, the prepared dry film was used for further analysis (Table 1 and Table 2).

### 2.3. Characterizations

#### 2.3.1. Analysis of Polymer Synthesis Results

The Fourier transform infrared spectrometer (FTIR) equipped with a DTGS detector (FT-IR-360, Perkin Elmer Enterprise Management Co., Ltd., Shanghai, China) was measured. The total attenuated reflectivity (ATR) technique is used for infrared measurement. The spectrum was obtained through 64 scans with a resolution of 2 cm^−1^, and a scanning range of 400–4000 cm^−1^ with a KBr pellet. All samples were dried in a vacuum oven at 40 °C for 48 h before testing.

The M_n_ values of MPEG-b-PLLA block copolymers were estimated by 600 MHz ^1^H-NMR spectroscopy (Bruker, Biospin, Switzerland), and CDCl_3_ and TMS were adopted as solvent and internal references, respectively.

The gel permeation chromatography (GPC) curve was measured by gel permeation chromatography (Malvern Viscotek 270, Worcestershire, UK), with N,N-dimethylformamide (DMF) used as a mobile phase at 40 °C. The sample was at a concentration of 1 mg/mL, with a flow rate of 1 mL/min and an injection volume of 100 μL. Characterization of the average molecular weight (Mn) and molecular weight distribution (Ð) was conducted via routine calibration using a refractive index (RI) detector and narrow distribution polystyrene (PS) as a standard.

#### 2.3.2. Wide Angle X-ray Diffraction (WAXD)

WAXD patterns of the samples were obtained using an X-ray diffractometer (Rigaku Corporation, Akishima, Japan) with Cu Κα radiation (λ = 0.154 nm) generated at 50 kV and 250 mA. Samples were scanned from 5° to 45° at a scanning rate of 5° min^−1^.

#### 2.3.3. Differential Scanning Calorimeter (DSC)

The DSC test was carried out using a Nash DSC-200F3 differential scanning calorimeter (NETZSCH-Ger ä tebau GmbH, Selb, Germany) equipped with an IC70 cooler under a nitrogen atmosphere. The samples were heated from room temperature to 200 °C at a rate of 10 °C min^−1^, and this was followed by heating for a duration of 5 min to eliminate any prior thermal effects. Subsequently, samples were cooled to 30 °C at a rate of 10 °C min^−1^ and then reheated to 200 °C at the same heating rate.

#### 2.3.4. Thermogravimetric Analysis (TGA)

The TGA test was conducted on a NETZSCH-209F1 thermogravimetric analyzer (NETZSCH-209 F1, Selb, Germany) equipped with a special IC40 cooler at a nitrogen flow rate of 100 mL/min. The sample was approximately 3–5 mg and was heated from 20 °C to 600 °C at a heating rate of 10 °C min^−1^.

#### 2.3.5. Polarized Optical Microscope (POM)

A polarizing microscope (POM) was used to observe the crystal morphology of spherulites. POM images were collected under a nitrogen atmosphere using a polarizing microscope (Eclipse E200 POL, Nikon, Japan) equipped with a hot table (LTS420; Linkam scientific instruments, Redhill, UK). The sample was melted at 180 °C, and the crystal morphology was observed at 120 °C. The overall crystallization behaviors of samples were also monitored using digital images.

#### 2.3.6. Environmental Scanning Electron Microscope (ESEM)

The morphology of the particles was monitored using a scanning electron microscope. These images were taken with an FEI Model Quantum 200 in high vacuum mode and an acceleration voltage of 15 kV. Using an ion coating device (FEI, Quanta 200, Hillsboro, OR, USA), the sample was plated with 20 nm thick electroplated gold.

## 3. Results and Discussion

### 3.1. Evaluation of the Preparation Parameters

FTIR and ^1^H-NMR spectra of Supplementary Material confirmed the synthesis of MPEG-b-PLLA material (Figures S1 and S2). As the molecular weight of the MPEG block was definite, the number average molecular weight of PLLA was determined based on the integral area ratio of 5.18 ppm (-CH-) and 3.66 ppm (-O-CH_2_-CH_2_-). The molecular weight is presented in Table 3.

The polydispersity index (PDI) of PEG, PDLA, and PLLA samples was determined via gel permeation chromatography (GPC). The specific data are shown in Table 4.

### 3.2. Crystalline Species and Crystallinity of MPEG-b-PLLA Copolymers, PDLA/MPEG-b-PLLA and PLLA/MPEG-b-PLLA Films

**Figure 2:**
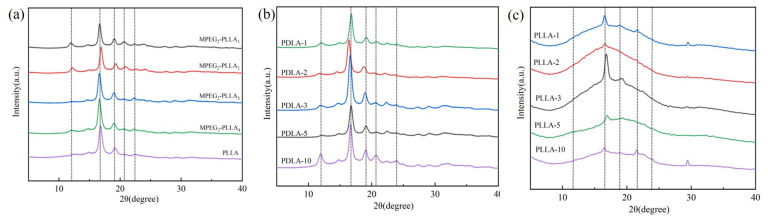
WAXD profiles of MPEG-b-PLLA diblock copolymers (**a**), PDLA/MPEG-b-PLLA composite film (**b**), and PLLA/MPEG-b-PLLA composite film (**c**) copolymers crystallized isothermally. The broken lines show the 2θ values.

In order to determine the crystal types of MPEG-b-PLLA diblock copolymers, WAXD measurements were conducted. The test results of copolymers and composite films are shown in Figure 2. PLA is recognized as a thermoplastic with relatively slow crystallization properties [38]. Pure PLA exhibits various polymorphs, including α, β, and γ forms. The α form is the most stable [39]. Through our experiments, we found that the MPEG-b-PLLA diblock polymer has a significant impact on the crystal structure of PDLA and PLLA blends. As shown in Figure 2, the diffraction peaks at 12°, 21°, and 24° correspond to the (110), (300/030), and (220) planes of stereocomposite crystals (SCs) [40,41]. The diffraction peaks at 16° and 19.0° correspond to the (110/200) and (203) planes of the α-form of homocrystals (HCs) [42,43]. The diffraction pattern in Figure 2 shows that the peaks related to the HCs coexist with SCs.

For the MPEG-b-PLLA diblock copolymer, all five peaks were observed, indicating that with the addition of MPEG, both stereocomplexes and homocrystals were observed in the polymer. For PDLA/MPEG-b-PLLA composite films, the peak values at 12° and 16° show a significant increase with an increase in MPEG-b-PLLA diblock copolymer content. This result indicates that: (1) For block copolymers, the introduction of PEG enhances HCs in the PLLA matrix, which is the role of melted PEG chain segments as the plasticizer and diluting agent [33]. In MPEG_2_-PLLA_1_ and MPEG_2_-PLLA_2_ diblock copolymers, the crystallization performs the best. (2) For composite films, PDLA-10 exhibits the strongest SCs, and PLLA-3 exhibits the strongest HCs.

### 3.3. Crystallization Behavior of PDLA/MPEG-b-PLLA and PLLA/MPEG-b-PLLA Composite Films

**Figure 3:**
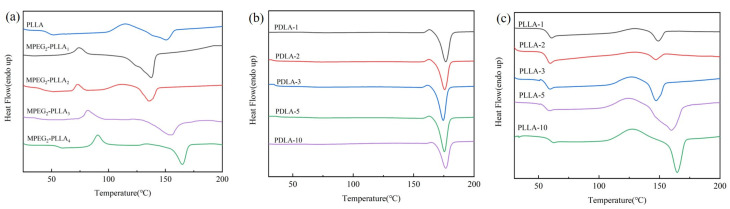
DSC thermograms of MPEG-b-PLLA and PLLA (**a**), PDLA/MPEG-b-PLLA (**b**) and PLLA/MPEG-b-PLLA (**c**).

Figure 3 shows the DSC curve of PDLA/MPEG-b-PLLA and PLLA/MPEG-b-PLLA composite films. The curves were analyzed, and we obtained the glass transition temperature (T_g_), cooling crystallization temperature (T_c_), melting temperature (T_m_), melting enthalpy (ΔH), and crystallinity (Χ_tc_) of PDLA/MPEG-b-PLLA and PLLA/MPEG-b-PLLA composite films. The T_g_, T_c_, and T_m_ of PLA films are 60.8, 134.3, and 159.5 °C, respectively. By calculating the ratio of the melting enthalpy of the sample to the melting enthalpy of fully crystallized PLLA (93.6 J/g) [44], we obtained the crystallinity of different samples.

For the PLLA/MPEG-b-PLLA composite films in Figure 3c, the addition of MPEG-b-PLLA block polymers had almost no significant effect on their T_g_, T_c_, and T_m_, but the melting enthalpy and crystallinity first increased and then decreased with an increase in MPEG-b-PLLA content.

**Figure 4:**
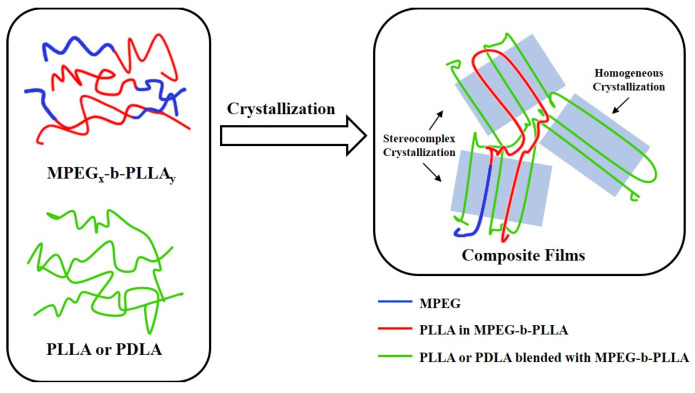
MPEG-b-PLLA and composite films crystallization principle.

Compared with the PLLA/MPEG-b-PLLA composite films, the T_c_ and T_m_ of the PDLA/MPEG-b-PLLA composite films all increased. The addition of MPEG-b-PLLA had a significant impact: (1) T_c_ first decreased and then increased. This is attributed to the plasticizing effect of the MPEG block [45], which is beneficial for reducing the conformational energy barrier and adjusting the regular stacking, and the PLLA chain was reset in order to be neatly stacked. As MPEG content increased, the early crystallization process α’ began. The increase in formation leads to an increase in T_c_. (2) α’-to-α [31,46]. The increase in the enthalpy value of the transformation indicates that during the early crystallization process, α’ increased crystallization; in other words, it enhanced crystallization performance. This can be attributed to the heat release signal of α’-to-α. Then, to a certain extent, the competitive effect between the MPEG and PLLA crystallization processes inhibited PLLA crystallization. Figure 4 provides a more intuitive representation of this principle.

Figure 3a and Table 5 show that the T_c_ of MPEG-b-PLLA samples is significantly reduced, as the addition of PEG acts as a plasticizer, promoting the migration of molecular chains, and crystallization can be completed at lower temperatures. Figure 3b and Table 6 show that the crystallinity of PDLA/MPEG-b-PLLA composite films increased significantly with the addition of MPEG-b-PLLA. Figure 3c and Table 7 show the crystallinity of the PLLA/MPEG-b-PLLA composite films increased first and then decreased with an increase in MPEG-b-PLLA addition. The results show that MPEG-b-PLLA acts as a nucleating agent in the process of the recrystallization of composite films and improves the crystallization ability of composite films [33]. It should be noted that in Table 6, the crystallinity of the PLLA-10 sample decreased, which may be due to the entanglement of the MPEG-b-PLLA block polymer with the pure polylactic acid chain, further reducing the segment’s movement [12].

### 3.4. Thermal Stability of MPEG-b-PLLA Diblock Copolymers

**Figure 5:**
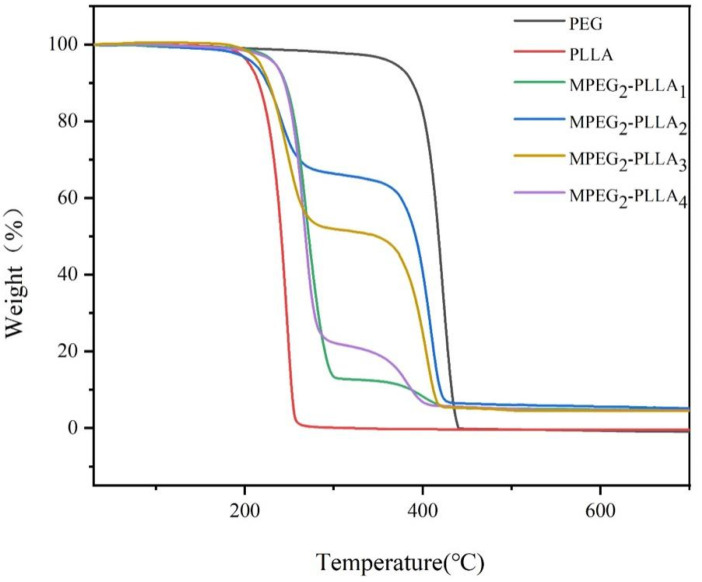
TGA curves of PEG, PLLA, and MPEG-b-PLLA.

TGA curves of PEG, PLLA, and MPEG-b-PLLA diblock copolymers with different chain lengths are shown in Figure 5. The weight loss of the test samples is 5% and 50%, and the maximum degradation rate temperature (T_max_) data are shown in Table 8. The thermogravimetric analysis results show that PEG and PLLA underwent a one-step thermal degradation process. PLLA starts to decompose at 170.6 °C and completes decomposition at 269.1 °C, with almost no residue. Due to its more stable ether bonds, PEG undergoes thermal decomposition at temperatures higher than PLLA, with an initial temperature of 352.2 °C and a stable temperature of 450.1 °C. The MPEG-b-PLLA diblock copolymer underwent a two-step thermal degradation process, and the starting temperature for thermal degradation is based on the intermediate values of MPEG and PLLA. The initial thermal degradation temperature (T_5%_) of MPEG_2_-PLLA_2_ has decreased, which may be due to the enhanced ester exchange effect of the PLLA segments at both ends under the low melting point MPEG, promoting the thermal degradation of the copolymer. However, for the copolymers of MPEG_2_-PLLA_1_, MPEG_2_-PLLA_3_, and MPEG_2_-PLLA_4_, there is a certain degree of increase in T_5%_ (~20 °C), which is explained by MPEG segments acting as plasticizers during the heating process to enhance the interaction (hydrogen bonding) between PLLA segments, thereby increasing the initial thermal degradation temperature (T_5%_). Meanwhile, the maximum thermal degradation rate temperature (T_1max_) and final decomposition temperature (T_final_) confirm the above results.

### 3.5. Morphological Structures of MPEG-b-PLLA Diblock Copolymers

**Figure 6:**
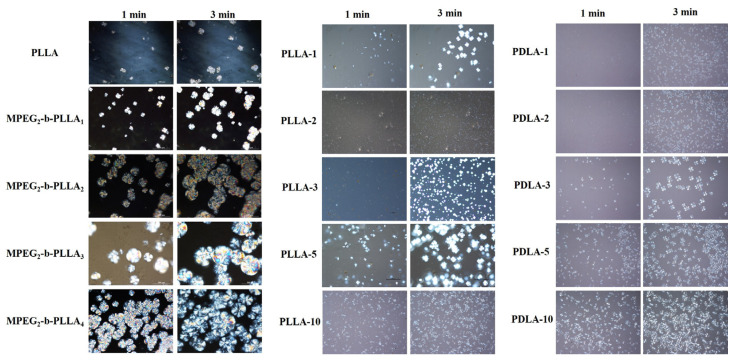
Isothermal crystallization of MPEG-b-PLLA diblock copolymer and composite film samples were observed under POM.

Polarization microscopy can be used to visually observe the spherical structure and growth behavior of MPEG-b-PLLA diblock copolymers. As shown in Figure 6, clear Maltese cross-shaped spherulites can be observed in all samples, indicating that the radial orientation of the SCs layer from the center of the spherulites is not affected by the chain segment’s length [33]. The higher the crystallinity, the stronger the crystallization ability and the faster the crystallization rate of SCs before HCs crystallization. Moreover, the number of spherical crystals per unit area is positively correlated with the molecular weight of the copolymer. The crystallization ability of the composite film is significantly enhanced with the addition of MEPG-b-PLLA.

**Figure 7:**
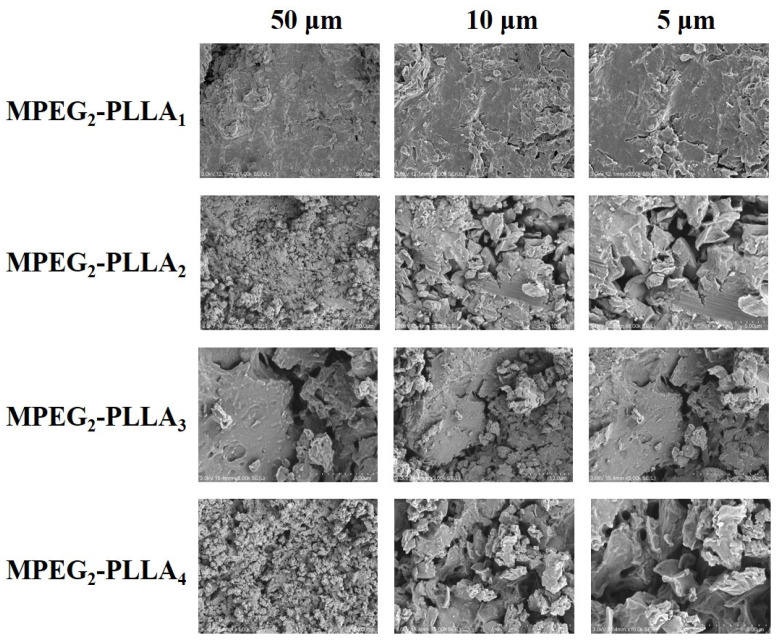
SEM images of MPEG-b-PLLA diblock copolymer samples at different magnifications.

**Figure 8:**
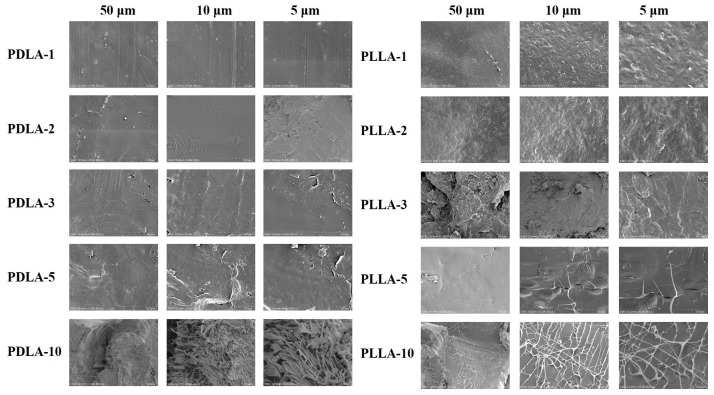
SEM images of PDLA/MPEG-b-PLA and PLLA/MPEG-b-PLA composite films at different magnifications.

In order to further explore the properties and characteristics of MPEG-b-PLLA copolymers, SEM analysis was chosen as a tool to directly observe the morphological differences between the particles of MPEG-b-PLLA copolymers with different structures. Figure 7 shows MPEG-b-PLLA copolymers with different surface structures. For the MPEG_2_-PLLA_1_ copolymer, its surface morphology and structure are relatively flat. The remaining sample images exhibit irregular concave–convex structures that have begun to appear in relatively dense surface morphology, with enhanced copolymer aggregation. In Figure 8, it can be seen that when the amount of MPEG-b-PLLA added is small, such as PDLA-1, PDLA-2, PLLA-1, and PLLA-2, the samples all exhibit a flat and smooth surface. As the content of MPEG-b-PLLA increases, irregular concave–convex structures appear on the surface of the composite film.

## 4. Conclusions

This article describes the use of WAXD, DSC, POM, and other methods to investigate the crystallization behavior and morphology of MPEG-b-PLLA and its composites with PDLA and PLLA. The results show that the introduction of MPEG accelerated the crystallization rate of PLLA, and the content of MPEG-b-PLLA had a significant impact on the crystallinity of PLLA and PDLA. The crystallinity of the composite film increases with an increase in block copolymer content. The isothermal crystallization of POM indicates that the addition of PEG promotes the crystallization of the polymer, and PEG chains have a nucleation effect on the formation of PLLA chain spherulites. During the heating process, PEG segments act as plasticizers to enhance the interaction between PLLA segments and form hydrogen bonds. In short, MPEG-b-PLLA copolymers regulate composites to achieve specific structural forms and offer potential insights for polymer medical materials, such as self-assembly.

## Figures and Tables

**Table 1:** Content of MPEG-b-PLLA in PLLA/MPEG-b-PLLA composite films.

Sample	PLLA-1	PLLA-2	PLLA-3	PLLA-5	PLLA-10
MPEG_2_-PLLA_2_ content (%)	1	2	3	5	10

**Table 2:** Content of MPEG-b-PLLA in PDLA/MPEG-b-PLLA composite films.

Sample	PDLA-1	PDLA-2	PDLA-3	PDLA-5	PDLA-10
MPEG_2_-PLLA_2_ content (%)	1	2	3	5	10

**Table 3:** HNMR results and component content of MPEG_x_-PLlA_y_.

Sample	PEG Unit Content (%)	PLLA Unit Content (%)	M_n_ (NMR) (g/mol)	M_n_ (GPC) (g/mol)	M_w_ (GPC) (g/mol)	PDI
MPEG_2_-PLLA_1_	46	54	4987	5532	5780	1.14
MPEG_2_-PLLA_2_	30	70	7792	8131	9113	1.12
MPEG_2_-PLLA_3_	22	78	7921	8294	8402	1.21
MPEG_2_-PLLA_4_	18	82	9856	10,088	10,110	1.16

**Table 4:** Molecular weights and polydispersity index of PEG, PDLA and PLLA.

Sample	M_p_ (g/mol)	M_n_ (g/mol)	M_w_ (g/mol)	M_z_ (g/mol)	M_z+1_ (g/mol)	M_v_ (g/mol)	PDI
PEG	2002	2009	2560	2801	2944	2412	1.20
PDLA	4215	4963	6317	7782	9961	5756	1.25
PLLA	4021	4652	6203	8576	11,382	5910	1.33

**Table 5:** Thermal properties of MPEG-b-PLLA diblock copolymers.

Sample	T_g_ (°C)	T_c_ (°C)	T_m_ (°C)	ΔH (J/g)	Χ(t) (%)
PLLA	60.8	134.3	159.5	13.9	14.8
MPEG_2_-PLLA_1_	-	80.8	142.4	17.6	18.8
MPEG_2_-PLLA_2_	-	79.6	143.9	17.3	18.5
MPEG_2_-PLLA_3_	-	92.3	163.2	13.6	14.5
MPEG_2_-PLLA_4_	-	97.7	171.3	12.1	12.9

**Table 6:** Thermal properties of PDLA/MPEG-b-PLLA composite films.

Sample	T_g_ (°C)	T_c_ (°C)	T_m_ (°C)	ΔH (J/g)	Χ(t) (%)
PDLA-1	-	163.5	176.3	9.5	10.1
PDLA-2	-	163.1	175.7	9.6	10.2
PDLA-3	-	161.9	174.3	14.5	15.5
PDLA-5	-	163.1	175.3	14.8	15.8
PDLA-10	-	165.5	176.5	17.0	18.2

**Table 7:** Thermal properties of PLLA/MPEG-b-PLLA composite films.

Sample	T_g_ (°C)	T_c_ (°C)	T_m_ (°C)	ΔH (J/g)	Χ(t) (%)
PLLA-1	60.7	130.4	148.8	8.4	9.0
PLLA-2	59.8	129.2	147.6	9.6	10.3
PLLA3	59.4	127.0	147.0	9.9	10.6
PLLA-5	59.2	124.1	160.3	11.2	12.0
PLLA-10	62.7	127.7	164.7	5.3	5.7

**Table 8:** Results of thermogravimetric analysis of PLLA, MPEG and MPEG-b-PLLA.

Sample	T_5%_ (°C) ^a^	T_50%_ (°C) ^b^	T_final_ (°C) ^c^	T_max_(°C) ^d^	
				Step1	Step2
PLLA	204.6	241.0	319.6	249.2	
MPEG	368.4	417.7	440.2	422.1	
MPEG_2_-PLLA_1_	234.9	271.8	487.9	267.2	400.5
MPEG_2_-PLLA_2_	195.2	392.0	424.2	240.1	408.5
MPEG_2_-PLLA_3_	233.9	268.1	434.8	268.6	379.5
MPEG_2_-PLLA_4_	217.1	348.2	419.4	249.0	404.9

^a^: Temperature at 5% weight loss. ^b^: Temperature at 50% weight loss. ^c^: The temperature at the time of final weightlessness. ^d^: Temperature at maximum degradation rate.

## Data Availability

Data are contained within the article or Supplementary Material.

## Supplementary Materials

The following supporting information can be downloaded at: https://www.mdpi.com/article/10.3390/ma17092105/s1, Figure S1: FTIR spectra of PLLA, MPEG-b-PLLA; Figure S2: ^1^H NMR spectra of MPEG-b-PLLA. References [24,47,48] are cited in the Supplementary Materials.

## References

[1] John R.P., Nampoothiri K.M., Pandey A. (2006). Solid-state fermentation for l-lactic acid production from agro wastes using *Lactobacillus delbrueckii*. Process Biochem..

[2] Chen C., Ding S., Wang D., Li Z., Ye Q. (2014). Simultaneous saccharification and fermentation of cassava to succinic acid by *Escherichia coli* NZN111. Bioresour. Technol..

[3] Carvalho J.R.G., Conde G., Antonioli M.L., Dias P.P., Vasconcelos R.O., Taboga S.R., Canola P.A., Chinelatto M.A., Pereira G.T., Ferraz G.C. (2020). Biocompatibility and biodegradation of poly(lactic acid) (PLA) and an immiscible PLA/poly(ε-caprolactone) (PCL) blend compatibilized by poly(ε-caprolactone-b-tetrahydrofuran) implanted in horses. Polym. J..

[4] Yan C., Hou D.-F., Zhang K., Yang M.-B. (2023). Effects of PDLA molecular weight on the crystallization behaviors and rheological properties of asymmetric PDLA/PLLA blends. Polymer.

[5] Zhang Y., Chen J., Fu Q., Zhang J. (2023). Novel Strategy to Improve the Performance of Poly(l-lactide): The Synergistic Effect of Disentanglement and Strong Shear Field. ACS Sustain. Chem. Eng..

[6] Shao J., Xu L., Pu S., Hou H. (2021). The crystallization behavior of poly(l-lactide)/poly(d-lactide) blends: Effect of stirring time during solution mixing. Polym. Bull..

[7] Wei Y., Tian Y., Tian X., Fu Z., Zhao L. (2022). Induction of Stereocomplex Crystallization in Poly(l-lactide)/Poly(d-lactide) Blends with High Molecular Weight by Halloysite Nanotubes. Macromol. Chem. Phys..

[8] Park H.-S., Hong C.-K. (2021). Relationship between the Stereocomplex Crystallization Behavior and Mechanical Properties of PLLA/PDLA Blends. Polymers.

[9] Lv T., Li J., Liu L., Huang S., Li H., Jiang S. (2023). Effects of molecular weight on stereocomplex and crystallization of PLLA/PDLA blends. Polymer.

[10] Liu X., Wang S., Huang K., Liu H., Zhang X., Zou L., Shi H., Chang B., Liu C. (2023). Competition effect of solid-state stretching induced orientation and phase separation on stereocomplex crystallization of PLLA/PDLA during annealing. Polymer.

[11] Li R., Wu Y., Bai Z., Guo J., Chen X. (2020). Effect of molecular weight of polyethylene glycol on crystallization behaviors, thermal properties and tensile performance of polylactic acid stereocomplexes. RSC Adv..

[12] Sun C., Zheng Y., Xu S., Ni L., Li X., Shan G., Bao Y., Pan P. (2021). Role of Chain Entanglements in the Stereocomplex Crystallization between Poly(lactic acid) Enantiomers. ACS Macro Lett..

[13] Li W., Srithep Y., Shen J., Pholharn D., Sriprateep K., Worajittiphon P., Khoklang N. (2024). Preferential formation of the stereocomplex crystals of poly(L-lactide) and poly(D-lactide) blend by epoxidized soybean oil under nonisothermal crystallization. Polym. Adv. Technol..

[14] Jing Z., Li J., Xiao W., Xu H., Hong P., Li Y. (2019). Crystallization, rheology and mechanical properties of the blends of poly(l-lactide) with supramolecular polymers based on poly(d-lactide)–poly(ε-caprolactone-co-δ-valerolactone)–poly(d-lactide) triblock copolymers. RSC Adv..

[15] Srithep Y., Pholharn D. (2017). Plasticizer effect on melt blending of polylactide stereocomplex. e-Polymers.

[16] Bai L., Zhao X., Bao R.-Y., Liu Z.-Y., Yang M.-B., Yang W. (2018). Effect of temperature, crystallinity and molecular chain orientation on the thermal conductivity of polymers: A case study of PLLA. J. Mater. Sci..

[17] Murthy N.S., Zhang Z., Borsadia S., Kohn J. (2018). Nanospheres with a smectic hydrophobic core and an amorphous PEG hydrophilic shell: Structural changes and implications for drug delivery. Soft Matter.

[18] Ju Z., Brosse N., Hoppe S., Wang Z., Ziegler-Devin I., Zhang H., Shu B. (2024). Thermal and mechanical properties of polyethylene glycol (PEG)-modified lignin/polylactic acid (PLA) biocomposites. Int. J. Biol. Macromol..

[19] Feng L., Ding J., Hu H., Lv Z., Zhang Y., Xu B., Quan J., Hao S., Fan H., Hang Z. (2023). Preparation and Characterization of Bio-Based PLA/PEG/g-C3N4 Low-Temperature Composite Phase Change Energy Storage Materials. Polymers.

[20] Nazari T., Bayandori Moghaddam A., Davoodi Z. (2019). Optimized polylactic acid/polyethylene glycol (PLA/PEG) electrospun fibrous scaffold for drug delivery: Effect of graphene oxide on the cefixime release mechanism. Mater. Res. Express.

[21] Kirmic Cosgun S.N., Ceylan Tuncaboylu D. (2021). Cyclodextrin-linked PVP/PEG supramolecular hydrogels. Carbohydr. Polym..

[22] Taipaleenmäki E.M., Mouritzen S.A., Schattling P.S., Zhang Y., Städler B. (2017). Mucopenetrating micelles with a PEG corona. Nanoscale.

[23] Zalba S., ten Hagen T.L.M., Burgui C., Garrido M.J. (2022). Stealth nanoparticles in oncology: Facing the PEG dilemma. J. Control. Release.

[24] Cui H., Shao J., Wang Y., Zhang P., Chen X., Wei Y. (2013). PLA-PEG-PLA and its electroactive tetraaniline copolymer as multi-interactive injectable hydrogels for tissue engineering. Biomacromolecules.

[25] Basu A., Kunduru K.R., Doppalapudi S., Domb A.J., Khan W. (2016). Poly(lactic acid) based hydrogels. Adv. Drug Deliv. Rev..

[26] Fujiwara T., Miyamoto M., Kimura Y. (2000). Crystallization-Induced Morphological Changes of a Poly(l-lactide)/Poly(oxyethylene) Diblock Copolymer from Sphere to Band via Disk:  A Novel Macromolecular Self-Organization Process from Core−Shell Nanoparticles on Surface. Macromolecules.

[27] Fujiwara T., Miyamoto M., Kimura Y., Sakurai S. (2001). Intriguing morphology transformation due to the macromolecular rearrangement of poly(l-lactide)-block-poly(oxyethylene): From core–shell nanoparticles to band structures via fragments of unimolecular size. Polymer.

[28] Sun J., Hong Z., Yang L., Tang Z., Chen X., Jing X. (2004). Study on crystalline morphology of poly(l-lactide)-poly(ethylene glycol) diblock copolymer. Polymer.

[29] Huang S., Jiang S., An L., Chen X. (2008). Crystallization and morphology of poly(ethylene oxide-b-lactide) crystalline–crystalline diblock copolymers. J. Polym. Sci. Part B Polym. Phys..

[30] Huang C.-I., Tsai S.-H., Chen C.-M. (2006). Isothermal crystallization behavior of poly(L-lactide) in poly(L-lactide)-block-poly(ethylene glycol) diblock copolymers. J. Polym. Sci. Part B Polym. Phys..

[31] Song Y., Wang D., Jiang N., Gan Z. (2015). Role of PEG Segment in Stereocomplex Crystallization for PLLA/PDLA-b-PEG-b-PDLA Blends. ACS Sustain. Chem. Eng..

[32] Guo M., Wu W., Wu W., Gao Q. (2022). Competitive Mechanism of Stereocomplexes and Homocrystals in High-Performance Symmetric and Asymmetric Poly(lactic acid) Enantiomers: Qualitative Methods. ACS Omega.

[33] Guo M., Zhao Z., Xie Z., Wu W., Wu W., Gao Q. (2022). Role of the Branched PEG-b-PLLA Block Chain in Stereocomplex Crystallization and Crystallization Kinetics for PDLA/MPEG-b-PLLA-g-glucose Blends with Different Architectures. Langmuir.

[34] Guo M., Wu W., Wu W., Wang R., Huang L., Gao Q. (2023). Recent advances in enhancing stereocomplexation between poly(lactide) enantiomeric chains. Phys. Chem. Chem. Phys..

[35] Shao J., Sun J., Bian X., Cui Y., Li G., Chen X. (2012). Investigation of Poly(lactide) Stereocomplexes: 3-Armed Poly(l-lactide) Blended with Linear and 3-Armed Enantiomers. J. Phys. Chem. B.

[36] Shao J., Xiang S., Bian X., Sun J., Li G., Chen X. (2015). Remarkable Melting Behavior of PLA Stereocomplex in Linear PLLA/PDLA Blends. Ind. Eng. Chem. Res..

[37] Pal A.K., Katiyar V. (2016). Nanoamphiphilic Chitosan Dispersed Poly(lactic acid) Bionanocomposite Films with Improved Thermal, Mechanical, and Gas Barrier Properties. Biomacromolecules.

[38] Zhao X., Yu J., Liang X., Huang Z., Li J., Peng S. (2023). Crystallization behaviors regulations and mechanical performances enhancement approaches of polylactic acid (PLA) biodegradable materials modified by organic nucleating agents. Int. J. Biol. Macromol..

[39] Tsuji H., Yamasaki M., Arakawa Y. (2021). Synthesis and Stereocomplexation of New Enantiomeric Stereo Periodical Copolymers Poly(l-lactic acid–l-lactic acid–d-lactic acid) and Poly(d-lactic acid–d-lactic acid–l-lactic acid). Macromolecules.

[40] Zhang B., Liu Y., Ren M., Li W., Zhang X., Vajtai R., Ajayan P.M., Tour J.M., Wang L. (2019). Sustainable Synthesis of Bright Green Fluorescent Nitrogen-Doped Carbon Quantum Dots from Alkali Lignin. ChemSusChem.

[41] Zhuang Z., Li T., Ning Z., Jiang N., Gan Z. (2022). Melt and nucleation reinforcement for stereocomplex crystallites in poly(l-lactide)/lignin-grafted-poly(ᴅ-lactide) blend. Eur. Polym. J..

[42] Sun Y., He C. (2012). Synthesis and Stereocomplex Crystallization of Poly(lactide)–Graphene Oxide Nanocomposites. ACS Macro Lett..

[43] Bao R.-Y., Yang W., Wei X.-F., Xie B.-H., Yang M.-B. (2014). Enhanced Formation of Stereocomplex Crystallites of High Molecular Weight Poly(l-lactide)/Poly(d-lactide) Blends from Melt by Using Poly(ethylene glycol). ACS Sustain. Chem. Eng..

[44] Zhu Q., Chang K., Qi L., Li X., Gao W., Gao Q. (2021). Surface Modification of Poly(l-lactic acid) through Stereocomplexation with Enantiomeric Poly(d-lactic acid) and Its Copolymer. Polymers.

[45] Hurst P.J., Rakowski A.M., Patterson J.P. (2020). Ring-opening polymerization-induced crystallization-driven self-assembly of poly-L-lactide-block-polyethylene glycol block copolymers (ROPI-CDSA). Nat. Commun..

[46] Xu C., Zhang J., Bai J., Ding S., Wang X., Wang Z. (2021). Two-Stage Crystallization Kinetics and Morphological Evolution with Stereocomplex Crystallite-Induced Enhancement for Long-Chain Branched Polylactide/Poly(D-lactic acid) Blends. Ind. Eng. Chem. Res..

[47] Gupta A., Mulchandani N., Shah M., Kumar S., Katiyar V. (2018). Functionalized chitosan mediated stereocomplexation of poly(lactic acid): Influence on crystallization, oxygen permeability, wettability and biocompatibility behavior. Polymer.

[48] Kim K.-S., Chung S., Chin I.-J., Kim M.-N., Yoon J.-S. (1999). Crystallization behavior of biodegradable amphiphilic poly(ethylene glycol)-poly(L-lactide) block copolymers. Appl. Polym..

