# Supplementary material for: Synthesis of MPEG-b-PLLA Diblock Copolymers and Their Crystallization Performance with PDLA and PLLA Composite Films
Source: Materials (Basel). 2024 Apr 29;17(9):2105. doi: 10.3390/ma17092105 (PMC11084635; doi:10.3390/ma17092105)
Supplement: Supplementary file 1 [file materials-17-02105-s001.zip › materials-2952479-supplementary.pdf]

1 Fourier Transform Infrared Spectrometer (FTIR)

The Fourier transform infrared spectrometer (FTIR) equipped with a DTGS detector was used for measurement. The total attenuated reflectivity (ATR) technique was used for infrared measurement. The spectrum was obtained through 64 scans with a resolution of 2 cm^{-1} and a scanning range of 400-4000 cm^{-1} with a KBr pellet. All samples were dried in a vacuum oven at 40 °C for 48 hours before testing.

FTIR spectroscopy is regarded as one of the techniques used for assessing changes in the conformations of films. As shown in Figure 1, the C=O stretching vibrations are represented at 1755 cm^{-1}. The bands at 2932, 2989, and 2853 cm^{-1} in response to the absorption of saturated C-H groups were more noticeable and wider than the control, demonstrating that the structure of PLA was modified by MPEG. In addition, compared with the control, the presence of the band at 3507 cm^{-1} could be attributed to the absorption of -OH and -COOH group; the presence of the bands at 1126 cm^{-1} and 1092 cm^{-1} (Figure S1a) could be attributed to the absorption of -C-O-C- group; the C-O stretching vibration is represented at 1300 cm^{-1}; and the C-H bending vibration is represented at 1400 cm^{-1}(Figure S1b), revealing the changed structure of PLA caused by the addition of MPEG[1].

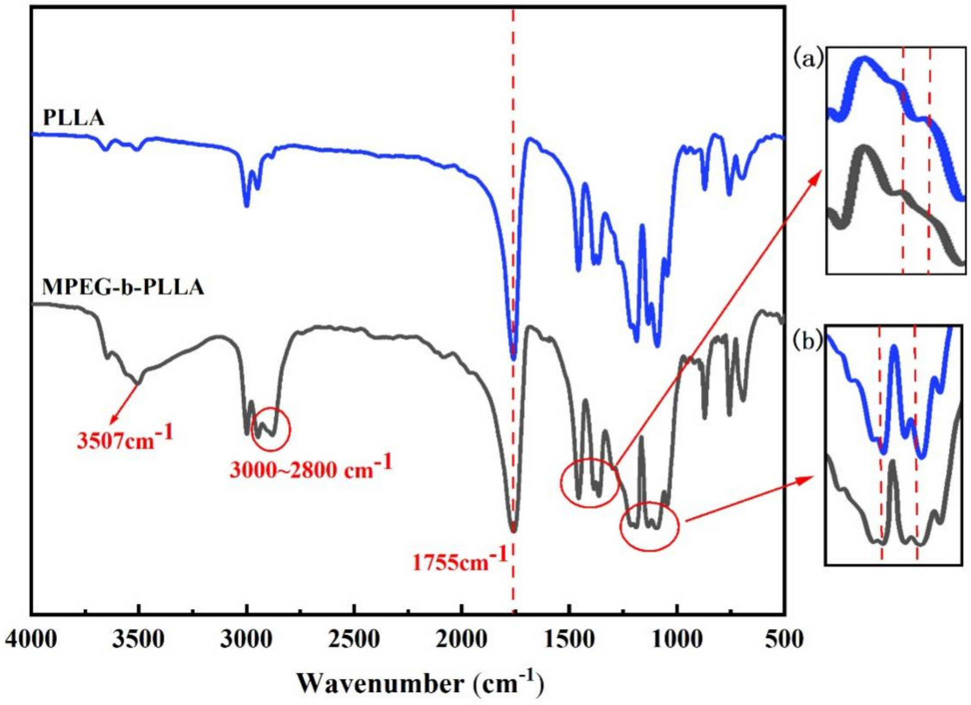

Figure S1. FTIR spectra of PLLA, MPEG-b-PLLA.

2 Nuclear Magnetic Resonance (NMR)

The M_n values of PLLA and MPEG-PLLA block copolymers were estimated by 600 MHz ^1H NMR spectroscopy (Bruker, Biospin, Switzerland), and $CDCl_3$ and TMS were adopted as a solvent and internal reference, respectively.

In the ^1H NMR spectrum in Figure S2, the signals located at 5.18 ppm (-CH-) and 1.58 ppm ($-CH_3$) were assigned to the PLLA block, and the peak at 3.66 ppm was ascribed to the signal of the MPEG block ($-O-CH_2-CH_2-$)[2,3]. As the molecular weight of the MPEG block was definite, the number average molecular weight of PLLA (M_n, PLLA) was determined based on the integral area ratio of 5.18 ppm (-CH-) and 3.66 ppm ($-O-CH_2-CH_2$).

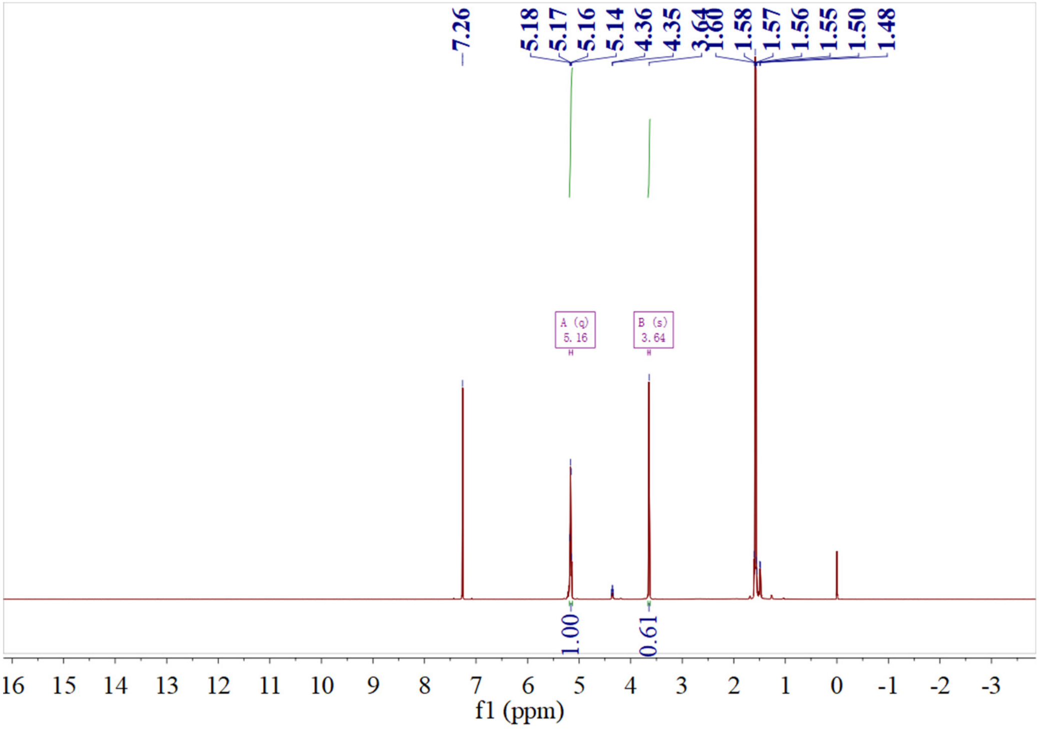

Figure S2. ^1H NMR spectra of MPEG-b-PLLA

References

1. Gupta, A., Mulchandani, N., Shah, M., Kumar, S., Katiyar, V., Functionalized chitosan mediated stereocomplexation of poly(lactic acid): Influence on crystallization, oxygen permeability, wettability and biocompatibility behavior. *Polymer* **2018,** *142*, 196-208.
2. Cui, H., Shao, J., Wang, Y., Zhang, P., Chen, X., Wei, Y., PLA-PEG-PLA and its electroactive tetraaniline copolymer as multi-interactive injectable hydrogels for tissue engineering. *Biomacromolecules* **2013,** *14* (6), 1904-12.
3. Kim, K.-S., Chung, S., Chin, I.-J., Kim, M.-N., Yoon, J.-S., Crystallization behavior of biodegradable amphiphilic poly(ethylene glycol)-poly(L-lactide) block copolymers. **1999,** *72* (3), 341-348.
